# Structure predicts function: Combining non-invasive electrophysiology with in-vivo histology

**DOI:** 10.1016/j.neuroimage.2014.12.030

**Published:** 2015-03

**Authors:** Saskia Helbling, Sundeep Teki, Martina F. Callaghan, William Sedley, Siawoosh Mohammadi, Timothy D. Griffiths, Nikolaus Weiskopf, Gareth R. Barnes

**Affiliations:** a Institute of Medical Psychology, Goethe University Frankfurt, Heinrich-Hoffmann-Str. 10, 60528 Frankfurt am Main, Germany; b Institute of Neuroscience, Newcastle University, Framlington Place, Newcastle-upon-Tyne NE2 4HH, UK; c Wellcome Trust Centre for Neuroimaging, Institute of Neurology, University College London, 12 Queen Square, WC1N 3BG London, UK; d Department of Systems Neuroscience, University Medical Center Hamburg-Eppendorf, Martinistraße 52, 20246 Hamburg, Germany

**Keywords:** MEG, Source reconstruction, Quantitative MRI, MPM, Myelin or myeloarchitecture, Auditory

## Abstract

We present an approach for combining high resolution MRI-based myelin mapping with functional information from electroencephalography (EEG) or magnetoencephalography (MEG). The main contribution to the primary currents detectable with EEG and MEG comes from ionic currents in the apical dendrites of cortical pyramidal cells, aligned perpendicularly to the local cortical surface. We provide evidence from an in-vivo experiment that the variation in MRI-based myeloarchitecture measures across the cortex predicts the variation of the current density over individuals and thus is of functional relevance. Equivalent current dipole locations and moments due to pitch onset evoked response fields (ERFs) were estimated by means of a variational Bayesian algorithm. The myeloarchitecture was estimated indirectly from individual high resolution quantitative multi-parameter maps (MPMs) acquired at 800 μm isotropic resolution. Myelin estimates across cortical areas correlated positively with dipole magnitude. This correlation was spatially specific: regions of interest in the auditory cortex provided significantly better models than those covering whole hemispheres. Based on the MPM data we identified the auditory cortical area TE1.2 as the most likely origin of the pitch ERFs measured by MEG. We can now proceed to exploit the higher spatial resolution of quantitative MPMs to identify the cortical origin of M/EEG signals, inform M/EEG source reconstruction and explore structure–function relationships at a fine structural level in the living human brain.

## Highlights

• We combined high-resolution MR-based myelin maps with functional MEG measures.
• Dipole moments of pitch onset responses correlated positively with myelin estimates.
• The correlation was spatially specific and most predictive for auditory areas.
• We can use MR-based myelin maps to identify the cortical origin of M/EEG signals.

## Introduction

A fundamental tenet of neuroscience is that function and structure are tightly linked. Early studies such as the work by Broca (1861) have demonstrated this relationship at a gross anatomical level. Invasive studies in animals clearly demonstrated that this relationship is not limited to the macroscopic level but also present at the microscopic level (Gibson et al., 2014). It has recently become possible to study the links between structure and function non-invasively using ultra high-resolution functional and anatomical magnetic resonance imaging (MRI) (e.g. Lutti et al., 2013; Yacoub et al., 2008). However, due to the dependence on the relatively slow haemodynamic response, functional MRI can only offer a rather indirect and temporally convoluted measure of function. Non-invasive electrophysiology based on magnetoencephalography (MEG) and electroencephalography (EEG) in contrast provide direct measures of cortical current flow at a millisecond time scale, but the spatial origins of the signals are generally less precisely known. In this study we show how information about myeloarchitecture (c.f. Vogt, 1909) from MRI can be used to refine source location estimates from MEG. It effectively combines the high spatial resolution and tissue microstructure specificity of MRI with the high temporal resolution of M/EEG.

EEG and MEG signals can be observed on the scalp due to the homogenous alignment and high local connectivity of pyramidal neurons in the cortical sheet generating a macroscopic net signal. The local connectivity means that excitatory drive will directly influence a large number of heavily connected local pyramidal cells (Holmgren et al., 2003; Schubert et al., 2007). The homogenous alignment of these cells means that their individual post-synaptic currents sum constructively giving rise to the M/EEG signal (Hämäläinen et al., 1993). Murakami and Okada (2006) estimated that a (typical) M/EEG current dipole moment of 10 nAm is due to the recruitment of around 50,000 pyramidal neurons. In this study we make use of the known microstructural variation in pyramidal cell density both across the cortex (von Economo, 1925) and over individuals and link these structural differences with the variation in cortical dipole moment strength as measured by M/EEG. Our motivating assumption here is that pyramidal cell density correlates positively with local myelin density in the cortex. Therefore we expect that MRI-based myelination measures would predict functional MEG measures.

This assumption is based on the close relationship between cyto- and myeloarchitecture: structural changes in cell density as revealed by Nissl stains tend to coincide with changes in intra-cortical myelinated fibres (Brodmann, 1909; Sanides, 1962). Hellwig (1993) quantified this relationship and translated cell densities into myelin maps by following two assumptions: (1) larger cells contribute more to the intra-cortical myelin content than small ones (represented by a sigmoid curve) and (2) the average distribution of horizontal axon collaterals of pyramidal cells can be quantified by a histogram of the number of these collaterals across layers (realised by a convolution in space with respect to the cell body).

Recent developments in MRI (Dick et al., 2012; Glasser and van Essen, 2011; Lutti et al., 2013; Sereno et al., 2013; Sigalovsky et al., 2006) have made it possible to non-invasively and indirectly measure myelin density throughout the cortex with high spatial resolution. MRI-based myelin mapping is based on measurements of physical relaxation times and magnetization transfer (MT) saturation. Quantitative multi-parameter mapping (MPM) has demonstrated robust differences in the longitudinal relaxation rate (R_1_) and MT saturation linked to myelin density differences between brain areas and across volunteers (Callaghan et al., 2014a). Dinse et al. (2013) developed a generative histology-based model of quantitative R1 contrast and used it to differentiate cortical functional areas based on their laminar R1 profiles. Similar results were found for the relation between the apparent transverse relaxation rate (R2*) and the myelin concentration (Cohen-Adad, 2014). The R1, R2* and MT values therefore provide non-invasive markers for myelination, and yield absolute MRI measures (Weiskopf et al., 2013), the latter being essential if we want to generally link neuromagnetic responses with myelination and systematically make inferences on structure–function relationships.

In this study we investigated whether the known variability in myeloarchitecture over individuals could predict the variation in dipole moment magnitude over individuals as estimated using MEG. Here, we do not intend to provide a comprehensive quantitative model describing the relationship between all functional, histological and MRI measures (please see Discussion), but rather aim to test whether such a relationship exists and to provide a rationale for how high-resolution MR-based measures can be exploited to refine our non-invasive electrophysiological estimates.

Features of the myeloarchitecture were estimated from individual high-resolution MPMs. We reanalysed data from a previous auditory MEG experiment (Sedley et al., 2012) where clear pitch-onset evoked response fields (ERFs) were found in the auditory cortex. ERFs reflect the summed neuronal activity across a large number of pyramidal cells and typically have an excellent signal-to-noise ratio. We hypothesised that subjects with higher myelin density in relevant auditory cortical areas would show larger equivalent current dipole moments when we source-localised the ERF peaks.

## Methods

### Auditory MEG experiment

We reanalysed data from a published MEG study on pitch perception and acquired quantitative multi-parameter maps for five of the subjects (mean age: 30.2 y, standard deviation (sd): 4.7 y, 3 female). The experimental paradigm is described in detail elsewhere (Sedley et al., 2012), and is summarised here briefly for convenience: pitch stimuli consisted of 1.2 s of regular interval noise (RIN) preceded immediately by 0.8 s of Gaussian noise. RIN was created from Gaussian noise using 16 iterations of an add-same process (Yost, 1996) with a delay of 1/256 s, corresponding to a pitch of 256 Hz. The transition from noise to RIN was associated with the onset of a pitch percept, but without any change in the gross power spectrum of the stimulus.

Subjects were passively exposed to the experimental stimuli, while fixating on a central point. Sounds were delivered diotically, via a pneumatic system and in-ear earmolds (Etymotic Research Inc.). There were 250 repetitions of each stimulus condition, presented in random order, with a fixed inter-stimulus interval of 1.5 s.

### MEG data acquisition and preprocessing

Data were acquired using a 275 channel whole-head MEG setup with synthetic third-order gradiometers (CTF systems) at 2.4 kHz sampling rate. Head location was determined using 3 fiducial markers, on the nasion as well as the left and right preauricular points. Data were converted for analysis in SPM12b (Wellcome Trust Centre for Neuroimaging; Litvak et al., 2011) and downsampled to 600 Hz. The data were divided into 3.5 s epochs spanning 0.75 s before to 2.75 s after stimulus onset. Any epochs containing peak-to-peak amplitude signals of greater than 5 × 10^− 12^ T were classified as artefacts and removed from the analysis (on average ~ 20% of trials per subject).

### Source reconstruction of evoked activity

Average waveforms were computed for all channels, low-pass filtered at 20 Hz and baseline corrected to the 100 ms period immediately preceding the noise-to-RIN transition. Equivalent current dipoles (ECDs) were fit to a single point in time corresponding to the peak of the pitch-onset response using a variational Bayes equivalent current dipole algorithm (VB-ECD; Kiebel et al., 2008). We employed a realistic single shell forward model (Nolte, 2003) based on the individual anatomical MRI of each subject. VB-ECD is a nonlinear optimization algorithm to fit a number of dipoles simultaneously to all of the sensor data at a single time point.

The difference between VB-ECD and other dipole fitting procedures is that the prior locations of the dipolar sources and their moments are treated as an explicit generative model of the data. This means we can use standard Bayesian methods and Bayesian model evidence to compare different models which might comprise different numbers of dipoles or dipoles with different priors. The model evidence is a balance between the accuracy of the fit to the data and the complexity of the model. The complexity of the model can be thought of as the flexibility of the model to explain any new data: in other words models with more dipoles or broader prior distributions will have more complexity.

We specified two independent ECDs for the whole brain, one for each hemisphere (left = [− 55.3; − 12.9; 1.5] mm and right = [57.2; − 8.8; − 1.3] mm in MNI coordinates), based on the contrast between fixed pitch and noise from a previous fMRI experiment (Patterson et al., 2002). Location priors were defined to have a standard deviation of 10 mm in each direction. Moment priors had a mean of 0 nAm and standard deviation of 100 nAm in the three cardinal directions. During the fitting procedure moment estimates were made in these three orthogonal directions for each source (i.e. there was no rank reduction). As MEG is relatively insensitive to the radial source component, after fitting this direction consequently has a very large, and uninformative, posterior variance (i.e. large uncertainty). We therefore determined the magnitude of the dipole moments from the projection onto the plane of the two smallest singular vectors (the most precise directions) of the moment estimates.

### MPM data acquisition

Data were collected on a 3 T whole body MR system (Magnetom TIM Trio, Siemens Healthcare, Erlangen, Germany) using the standard 32-channel radio-frequency (RF) receive head coil and RF body coil for transmission. A whole-brain, quantitative multi-parameter mapping protocol (Dick et al., 2012; Sereno et al., 2013; Weiskopf et al., 2013) was used to acquire 800 μm isotropic resolution brain images. The protocol comprised three RF spoiled 3D multi-echo FLASH scans with predominantly proton density (PDw: repetition time TR = 25.25 ms; flip angle α = 5°), T1 (T1w: TR = 25.25 ms; α = 29°) and magnetization transfer (MTw: TR = 29.25 ms; α = 9°) weightings. Eight alternating gradient echoes were acquired at equidistant echo times (from 2.39 to 18.91 ms, spacing: 2.36 ms) for the T1w, MTw sequences, and the PDw sequence (matrix = 320 × 280 × 208 pixels; FoV = 256 × 224 × 167 mm; GRAPPA factor 2 in phase encoding direction and 6/8 Partial Fourier factor in partition direction). In addition, a 3D EPI acquisition of spin and stimulated echoes with 15 different refocusing flip angles (TE/TM/TR = 39.38/33.24/500 ms; matrix = 64 × 48 × 48 pixels; FoV = 256 × 192 × 192 mm) was acquired to estimate the local RF transmit field (Lutti et al., 2010, 2012). A B_0_ field-map was acquired using a double-echo gradient echo sequence (TE1 = 10 ms, TE2 = 12.46 ms, TR = 1020 ms, 3 × 3 × 2 mm resolution, 1 mm gap; matrix size = 64 × 64 pixels; FoV = 192 × 192 × 191 mm) to allow for post-processing correction of geometric distortions of the EPI data due to B_0_ field inhomogeneity. Total acquisition time for all MRI scans was about 36 min.

### Estimation of parameter maps

MPM maps were estimated from the multi-echo FLASH scans using the VBQ toolbox (Draganski et al., 2011) with B1 mapping data in SPM8. Quantitative R1 maps were estimated from the PDw and T1w images according to the model developed by Helms et al. (2008) including a correction for RF transmit field inhomogeneities (Lutti et al., 2010) and imperfect RF spoiling (Preibisch and Deichmann, 2009). Correcting for B1 field inhomogeneities is essential as even modest spatial RF biases can mask the small myelin-related signal changes (Lutti et al., 2013). MT maps were estimated from all three multi-echo FLASH scans according to Weiskopf et al. (2013) and included a higher order RF bias correction. The apparent transverse relaxation rate R2* was estimated from the logarithm of the signal intensities (from the 8 PDw multi-echo images) at different echo times using a linear regression (Weiskopf et al., 2014).

MT maps were classified into grey matter (GM), white matter (WM) and cerebrospinal fluid (CSF) using a Gaussian mixture model within the unified segmentation approach (Ashburner and Friston, 2005). DARTEL processing (Ashburner, 2007) was used to spatially normalise individual grey and white matter images to a group mean template image in standard MNI space generated from the structural MT maps. MT maps have similar WM/GM contrast in the cortex as T1w images, but have significantly improved contrast in subcortical structures (e.g. basal ganglia, see Helms et al., 2009). Images were smoothed using a 3 mm full-width at half-maximum (FWHM) Gaussian smoothing kernel. The spatial normalisation and smoothing process included the correction for partial volume effects and preservation of quantitative values as implemented in VBQ (Draganski et al., 2011).

### Masking and defining regions of interest

Grey and white matter probability maps based on the MT map were transformed to MNI space based on the DARTEL template, modulated with the Jacobian determinant and smoothed with an isotropic Gaussian kernel of 3 mm FWHM. Smoothed MT-derived GM images were masked at a probability of 0.2 for each subject. WM masks were defined more strictly by thresholding the maps at a probability of 0.8 of WM, again for each subject individually.

**Fig. 1:**
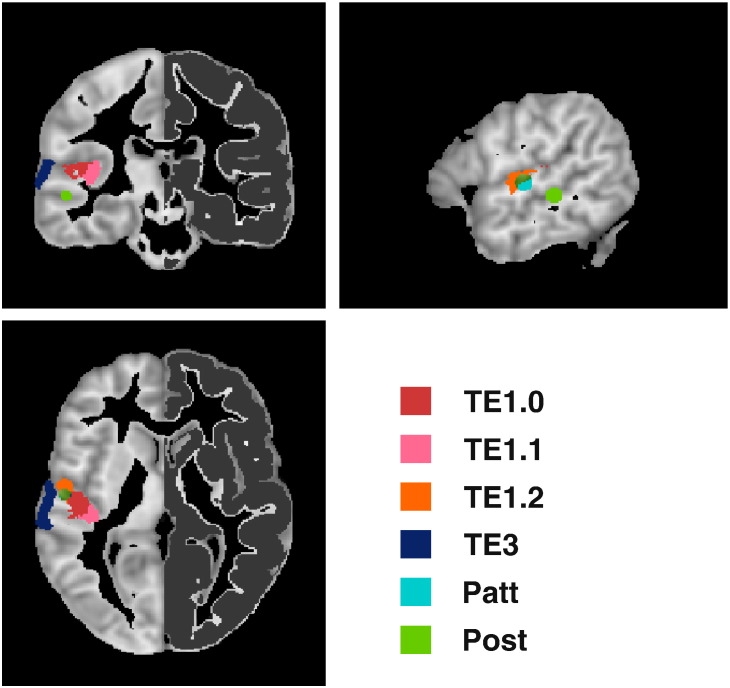
Regions of interest used to extract the quantitative MPM values. Four anatomically defined regions of interest from the Anatomy toolbox were used: auditory koniocortex areas TE1.0, TE1.1 and TE1.2 and secondary auditory cortex area TE3, as described in Morosan et al., 2001. Additionally, we defined two spherical ROIs with a radius of 5 mm centred around the prior locations used in the VB-ECD algorithm (*Patt*) and around the mean posterior locations (*Post*). ROIs were further masked with a grey matter mask as shown here in dark grey for the right hemisphere.

We used four anatomically defined regions of interest from the Anatomy toolbox (Eickhoff et al., 2005): auditory koniocortical areas TE1.0, TE1.1 and TE1.2, with a prominent layer IV, and auditory cortex area TE3, at the superior temporal gyrus as described in Morosan et al., 2001. TE1.1, TE1.0 and TE1.2 are located at Heschl's Gyrus (HG), the site of the primary auditory cortex, where TE1.1, TE1.0, and TE1.2 can be identified along the mediolateral axis. Additionally, we defined spherical ROIs with a radius of 5 mm centred around the coordinates of the location priors based on the study by Patterson et al. (2002) used in the VB-ECD source localisation (here labelled *Patt*) and around the resulting mean posterior locations in the left ([− 54.3; − 30.0; − 6.1] mm) and right ([56.9; − 28.6; − 13.3] mm) hemisphere (labelled *Post*). ROIs were masked with the GM mask for each individual (see Fig. 1). Quantitative MRI values were averaged across GM-masked ROIs for each of the three different MPMs (R1, MT, R2*).

### Statistics

#### Testing for differences in myelination between auditory areas

We determined the inter-areal variability of the anatomy by means of one-way repeated-measures ANOVAs with factor ROI (levels: TE1.0, TE1.1, TE1.2, TE3, Patt and Post) across hemispheres for the different quantitative MPMs. To correct for multiple comparisons across multi-parameter maps, we performed a Bonferroni correction resulting in an alpha level of 0.05/3 = 0.0167 for each MPM.

Post-hoc tests comprised two-sided t-tests, with an alpha level p < 0.05/15 = 0.0033 (Bonferroni-corrected across comparisons).

#### Covariation between MPM and MEG data

We tested whether increased myelination measured by MPM correlated with increased dipole moments measured by MEG. Due to the small number of subjects in this pilot study, we treated each hemisphere independently to increase statistical power (n = 10).

As we had three MPM metrics and a set of six cortical areas of interest we first set out to verify that any overall significant correlation between myelin estimates and dipole moments was not due to chance. This provided us with 18 Spearman's correlation coefficients which we summed to get a single metric across auditory areas of interest and MPMs. We then estimated the null distribution of this sum (in the case of no correlation this should tend to zero) using permutation testing. We randomly re-assigned hemisphere labels to obtain the distribution of the sum of correlation coefficients under the null-hypothesis of no correlation between dipole moments and myelin estimates. The sum of correlation coefficients based on the original (unpermuted) data set was then compared to the empirical distribution based on 5000 permutation sets.

The specificity of the structure–function relationship was further investigated by testing whether myelin estimates are spatially specific in predicting dipole moments. We used variational Bayes General Linear Models (GLMs) to compare three regions of interest differing in their spatial extent: a sub-region of the auditory koniocortex (TE1.2), the whole of the auditory koniocortex TE1 (including TE1.1., TE1.0 and TE1.2) and a third model in which we take the whole hemisphere. Here, dipole moments served as dependent variables, while myelin estimates at each ROIs of the different quantitative maps acted as explanatory variables in the design matrix. Bayesian GLMs return a variational approximation to the model evidence (Friston et al., 2002a, b) and are penalised by model complexity (i.e. the model evidence begins to decrease once the regression becomes over parameterised). A difference in log model evidence of more than 3 means that one model is approximately 20 (1 / (1 + exp(3))) times more likely than another (Kass and Raftery, 1995).

In order to determine which of the six ROIs and which myelin map predicted the dipole moments best we used family model comparison (Penny et al., 2010) and either factored out the type of MR-based myelination estimate and made inference on regions; or factored out ROIs and made inference on the most useful MR-based myelination estimate. In both cases we assumed a uniform prior probability of each map (i.e. 1/3 for each of the 3 maps) and each ROI (i.e. 1/6 for each of the 6 ROIs).

## Results

### Pitch-onset evoked response fields

**Fig. 2:**
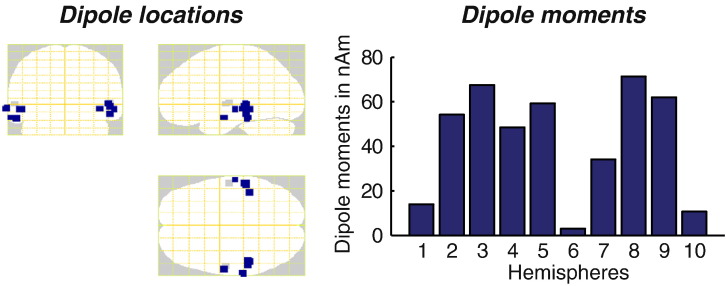
Locations of the optimised coordinates and respective dipole moments yielded by the VB-ECD source reconstruction across subjects. Left panel: optimal source locations for each participant plotted on a glass brain in MNI space. Location priors are shown in grey and were based on a previous MRI study by Patterson et al., 2002 (left hemisphere: [− 55.3; − 12.9; 1.5] mm and right hemisphere: [57.2; − 8.8; − 1.3] mm). Right panel: dipole moments in nAm across the ten hemispheres.

The mean latency of the ERF peak after the transition to RIN was 147.7 ms (sd: 9.3 ms), in line with previous studies of pitch-onset responses (Krumbholz et al., 2003). In all cases there was a clear best fitting two-dipole model at the peak ERF latency which was reproducible across re-initialisations (different starting points within the prior distributions) of the VB-ECD source reconstruction procedure. ECDs explained more than 95% of the sensor level variance for all subjects. Posterior locations in MNI space were mean (sd) = [− 54.3 (8.1); − 30.0 (8.1); − 6.1 (5.6)] mm for the left and [56.9 (7.7); − 28.6 (12.4); − 13.3 (6.5)] mm for the right hemisphere. Dipoles were all located in auditory areas (Fig. 2, left panel). Dipole moments varied across hemispheres and spanned a range from 3.01 to 71.34 nAm (mean (sd): 42.46 (25.27 nAm)) as shown in the right panel of Fig. 2. Visual inspection verified that the dimension-reduced dipole moments were oriented tangentially to the cortical surface.

### Mean MPM values across grey and white matter

Quantitative values of the MT, R1 and R2* maps were averaged across grey and white matter separately. Mean MPM values for GM were 0.8521 p.u. for the MT saturation, 0.628 s^− 1^ for R1 and 19.0 s^− 1^ for R2* and WM values were 1.6855 p.u. for MT, 0.944 s^− 1^ for R1 and 20.0 s^− 1^ for R2*.

**Fig. 3:**
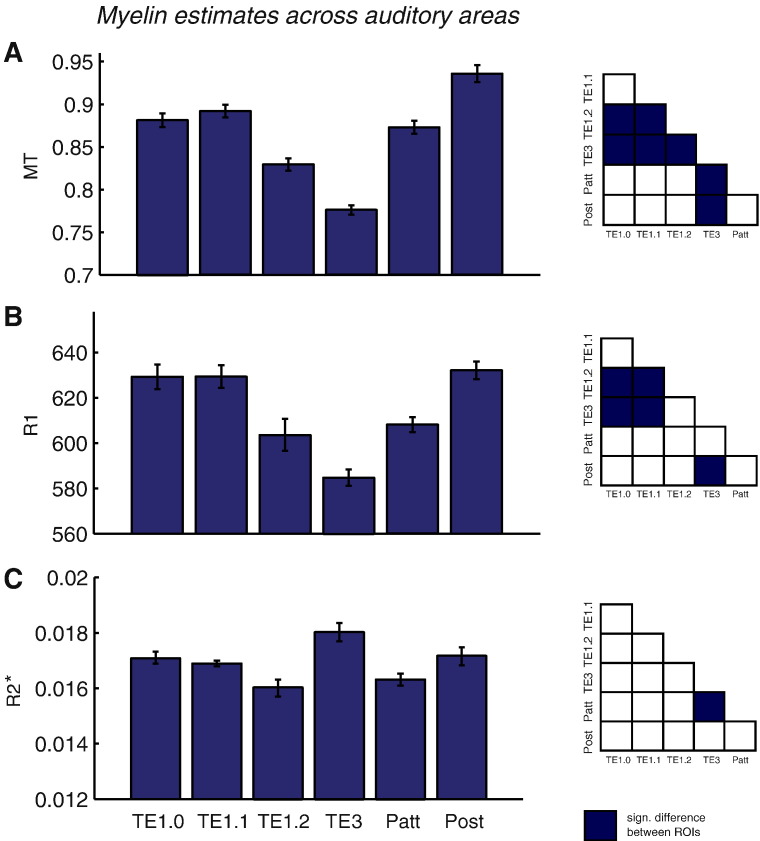
Myelin estimates across auditory regions of interest for A the magnetization transfer (MT) map, B the R1 map and C the R2* map. Error bars denote standard error of the mean. Statistically significant differences between pairs of ROIs are indicated in dark blue on the small schemas next to each bar plot.

In general, we found that myelination estimates were larger in core koniocortical regions TE1.1 and TE1.0 than in the more transitionary area TE1.2 or secondary auditory cortex area TE3 (for MT and R1 maps). Results are summarised in Fig. 3.

### Dipole moments correlate positively with myelination estimates

Table 1 lists the Spearman's r correlation coefficients between the different MPM maps and dipole moments over ROIs and hemispheres. Note that all the coefficients are positive. We first tested the likelihood of this relationship having arisen by chance, and then examined its spatial specificity.

**Fig. 4:**
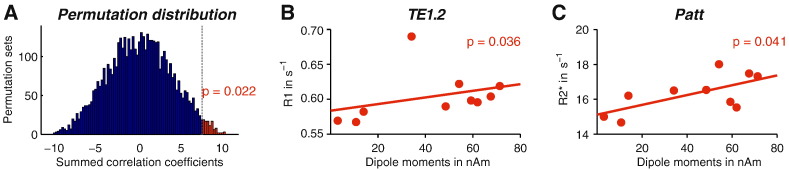
Significant correlations between dipole moments and myelin estimates. A. Distribution of Spearman's r correlation coefficients summed over ROIs and quantitative MPMs for 5000 permuted data sets. The observed value of summed r's = 7.58 (dashed grey line) for the original data set was exceeded by the permutation distribution values indicated in red, yielding a p-value of 0.022. B. Quantitative R1 values averaged across TE1.2 and C. R2* values averaged across *Patt* correlated positively with dipole moments.

To control for the inflated family wise error (FWE) rate from the multiple comparisons we performed a permutation test by randomly permuting the ten hemisphere labels to create an empirical null distribution of the sum of the correlation values. The permutation test revealed a significant positive relationship between myelination estimates of auditory regions and pitch-onset response dipole moments (summed r's = 7.58; p < 0.05, FWE corrected, one tailed; Fig. 4 A). Figs. 4B, C show scatter plots of dipole moments over hemispheres against corresponding ROI specific MPM metrics for the two regions that showed the largest correlation with the dipole moments.

Dipole moments correlated positively with R1 map-based myelin estimates of auditory area TE1.2 (r = 0.600; p < 0.05) and with R2* map-based myelin estimates of the spherical ROI *Patt* (r = 0.588; p < 0.05). The corresponding regression slopes were 0.475 s^− 1^ per nAm for R1 (intercept at 0.580 s^− 1^) and 0.281 s^− 1^ per nAm for R2* (intercept at 15.1 s^− 1^).

**Fig. 5:**
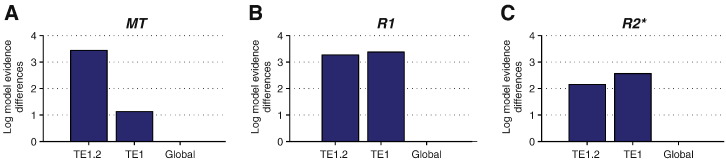
Spatial specificity of the positive relationship between myelin estimates and dipole moments. Variational Bayes GLMs with myelin estimates based on A the MT, B the R1 or C the R2* maps were used as independent variables to explain dipole moments across ROIs with different spatial extent. Differences in log Bayes factors for ROIs with increasing spatial extent, TE1.2, auditory koniocortex TE1 (i.e. TE1.0, TE1.1 and TE1.2 combined) and myelin estimates averaged across the whole hemisphere (*global*), are displayed. Since the log model evidence is a relative metric, all plots are normalised by the worst model of the corresponding quantitative MPM (set to zero). Log Bayes factor differences larger than 3 indicate that the data support the model with the larger Bayes factor.

Fig. 5 shows that this positive correlation was due to local changes in myelination. The spatial specificity can be observed for all MPM metrics, with significant (log model evidence > 3) improvement over the global model in TE1.2 and auditory koniocortex TE1 for the R1 and at TE1.2 for the MT map. The hemisphere sized ROI was the worst model for all MPMs, ruling out potential confounds such as gross measures of myelination or brain size.

**Fig. 6:**
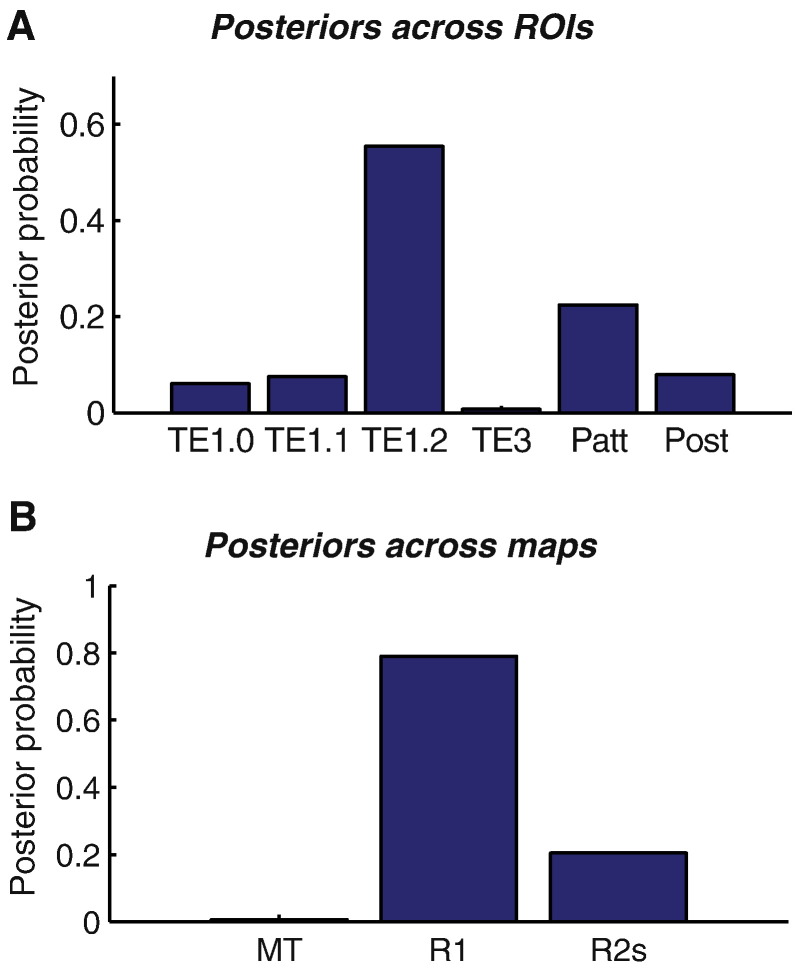
Posterior probabilities of Bayesian model comparison families for A ROIs and B myelination maps. Overall, the MRI-based myelin measures predict the dipole moments best in TE1.2. The R1-based myelin estimates predict MEG dipole moments best across all ROIs.

We further extended this model comparison across the six sub-regions and 3 MPM maps. This gave 18 model evidence values for the different map and ROI combinations. We used family model comparison (Penny et al., 2010) and marginalised either across MPMs or across ROIs. As shown in Fig. 6, based on this family comparison, the most likely origin of the MEG evoked response was TE1.2 with a probability of 0.5548. Marginalising over ROIs (Fig. 6B) suggested that R1 myelin maps were the most useful (p = 0.7897) predictors of dipole moment.

## Discussion

We have demonstrated that non-invasive MR-based estimates of the local myelin density predict dipole moment magnitudes, and that this information is spatially specific. This link opens up a number of exciting possibilities in the exploration of structure–function relationships in the human brain. In particular, the high spatial specificity of the MPM measures can be combined with the high temporal resolution of M/EEG.

### MRI-based myelination measures reflect key findings of ex vivo myelo- and cytoarchitecture

The main reason that we were able to link function to anatomy was the quantitative nature of the MPM maps used as myelin density estimates. Mean MPM values across grey and white matter corresponded well with values reported in previous studies (Baudrexel et al., 2009; Weiskopf et al., 2013; Wright et al., 2008), demonstrating their robust reproducibility. Further, we were able to reproduce in vivo previous findings from ex vivo histology by showing that auditory koniocortical core regions TE1.0 and TE1.1 are more heavily myelinated than TE1.2, while region TE3 is less myelinated than TE1 (Morosan et al., 2001), as reflected in the R1 and MT values. Cytoarchitectonic studies in humans indicate that area TE1.2, located at the lateral HG, is less ‘primary-like’ than medial and middle HG and constitutes a ‘transition zone between primary and non-primary areas’ (von Economo, 1925; Morosan et al., 2001). Previous studies using MR-based myelin estimates also found auditory core areas to be more heavily myelinated than more lateral areas (Dick et al., 2012; Sigalovsky et al., 2006). Functionally, this matches the results of a dynamic causal modelling (DCM) study by Kumar et al. (2011) who found that the lateral HG is at a higher level of hierarchy than medial and middle HG.

Surprisingly, we also found high myelin estimates for the spherical ROI *Post* which lies outside the highly myelinated koniocortex. We cannot exclude that the apparently high myelin estimate was caused by a partial volume effect between grey and white matter (with a high myelin concentration), since for the *Post* ROI the demarcation of white matter was more complicated than for the other ROIs. The *Post* ROI overlapped to a large extent with the smoothed WM image of the MT map and thus the distinction relied strongly on an accurate grey matter mask.

### Quantitative multi-parameter maps as biomarkers for myelination

It is not yet established which MPM map is most informative of myelin content. Myelin is widely accepted to contribute largely to measured MT effects in the brain (Bjarnason et al., 2005; Schmierer et al., 2004; Stanisz et al., 1999). The particular MT saturation measure used in this study is to a high degree insensitive to T1- and T2*-weighting effects and RF transmit field inhomogeneities (Weiskopf et al., 2013). Thus, it is a more specific marker for myelination than the conventional MT ratio measures (Callaghan et al., 2014b). Quantitative R2* maps reflect mainly iron content (Stüber et al., 2014; Langkammer et al., 2010) while R1 maps correlate positively with myelin concentration, but also with iron content (see Callaghan et al., 2014a, 2014b). Iron was found to be involved in myelination repair and maintenance as well as in lipid synthesis (Fukunaga et al., 2010; Todorich et al., 2009). Iron and myelination have been shown to be co-localised in the human brain by a recent susceptibility mapping study exploring the cortical myeloarchitecture (Fukunaga et al., 2010). Recent studies suggest a linear relationship between myelination and iron concentration on the histological side and R1 and R2* on the MR imaging side (Callaghan et al., 2014b; Stüber et al. 2014). Here, marginalising over regions, we found the R1 map to be the most likely predictor of dipole moment (Fig. 6B). The higher predictive power may be due to the relatively high SNR of this map and the synergistic effect of myelin and co-localised iron content.

### Refining M/EEG source localisation

The use of the myelin density maps, and their substantial inter-regional variability (Fig. 3), adds an extra dimension with which to refine our inference on the sources of these auditory evoked responses. The VB-ECD dipoles (cmp. Results 3.1) were located more posteriorly and inferiorly to the priors derived from Patterson et al., 2002 (cmp. Methods 2.3). After the initial dipole fits, the standard deviation of these source locations in each principal direction was of the order of 1 cm. This was in line with what one might expect from conventional co-registration procedures (Singh et al., 1997; Stolk et al., 2013). Based on the covariation of dipole moment with myelination (marginalised over maps) we refined our source localisation and found the most likely origin of the MEG signal to be area TE1.2 (followed by *Patt* ROI), located in the lateral third of HG. The spherical *Patt* ROI is close to TE1.2, partly overlapping with it and extending towards more medial areas. Importantly, we were also able to reject the possibility that this relationship could be explained by any global changes in myelination over hemispheres (Fig. 5).

A number of previous fMRI studies (Griffiths et al., 1998; Penagos et al., 2004; Patterson et al., 2002; Puschmann et al., 2010) have emphasised a role for lateral HG in the processing of temporal regularity and the perception of pitch (although some activity occurs in more medial areas too). An MEG study by Gutschalk et al. (2004) used pitch-evoking click-trains and source localised the pitch-onset responses to lateral HG. Krumbholz et al., 2003, in another MEG study, employed RIN stimuli very similar to the ones used in the present work and identified medial HG as the origin of the pitch-onset response. Responses to temporally regular sounds, when these have rates associated with pitch, were reported in human primary cortex in the medial half of HG and adjacent non-primary areas in lateral HG by direct recordings of local field potentials (Griffiths et al., 2010). In summary, our results correspond well with previous studies on auditory perception of pitch, where lateral HG is frequently found to be involved in pitch processing but other auditory areas, particularly the medial half of HG, were also reported. With regard to the question of whether medial or lateral HG is the more likely origin of the pitch-onset response, incorporating myelin density estimates pointed to lateral HG. Given the small number of subjects in the study, our finding of TE1.2 certainly needs to be replicated to provide compelling evidence for this auditory sub-area being the origin of the pitch-onset response. Here we wanted to provide a proof-of-principle for how we can refine source localisation estimates by using myelin density estimates.

An important potential confound of our study is the natural variation in extent, shape and orientation of the auditory cortex across individuals (e.g. Rademacher et al, 2001). One way around this would be to define auditory sub-regions on an individual basis. For example, Dick et al., 2012, were able to identify auditory core areas by combining laminar profiles of quantitative R1 maps with functional tonotopy. In the present study we cannot draw on functional localisers, nor did we estimate laminar profiles, but future studies might benefit from individually defined ROIs based on quantitative MPMs.

For our present purpose we would like to note that the average quantitative MPM values are in accordance with both previous post-mortem cytoarchitectonic analyses (Morosan et al., 2001) and recent MR-based in-vivo histology studies (Dick et al., 2012), which showed higher cell volume density and myelination, respectively at auditory core areas than at the neighbouring, less primary-like areas TE1.2 and TE3 (Fig. 3). This reassured us that there is good correspondence between the probabilistically defined ROIs and the actual cortical regions. Although we cannot rule out that this correlation was in some way driven by other factors, these factors could be expected to rather introduce noise that would confound rather than support our hypothesis. For example, when the regional templates are based on probabilistic atlases it is possible that between subject differences in the relative size of an area could bias the myelin estimates and/or decrease spatial precision.

### Relating myelination to functional measures

There are a number of other ways, besides reflecting a larger pyramidal cell density, in which a local increase in myelination might be linked to the MEG signal. For example, one factor might be that more heavily myelinated regions index higher densities of larger relative to smaller neurons (e.g. layer 5 pyramidal cells compared to layer 2/3 pyramidal cells). In this case the longer cell bodies will give rise to an increased dipole moment (for the same current per unit area) (Jones et al., 2007; Murakami and Okada, 2006). Another factor might be that neurons with denser collateral connections are likely to produce current distributions which extend over larger cortical areas. If this activity is modelled as a dipolar source (with no area) the effective dipole moment would also be increased.

Structure–function relationships in the auditory domain are well-known and have been reported previously (Hall et al., 2003; da Costa et al., 2011). Notably, Schneider et al. (2002) demonstrated that the dipole moment of early neurophysiological source activity in the auditory cortex evoked by sinusoidal tones was strongly correlated with the mean grey matter volume of anterior-medial HG. Our study moves beyond these findings by considering not only the shape of cortical areas, but also their local myeloarchitecture. Morphometry and measures of myeloarchitecture can complement each other: Freund et al. (2013) were able to show that while changes in WM and GM volume of the spinal cord and the brain correlated with clinical outcome in patients with chronic spinal cord injuries, the same was true for changes in myelin-sensitive MR-parameters, at the same areas and beyond. Importantly, incorporating these myelin-sensitive quantitative MPMs can enable us to reveal the mechanisms underlying macroscopic changes, i.e. the findings of Freund et al. (2013) support the hypothesis that the loss of myelinated axons within the corticospinal tracts leads to the macroscopic volume loss detected by VBM.

### Future directions

Now that a quantitative relationship between myelin-sensitive MPM metrics in auditory cortex and dipole moment has been established, one could use the regression slopes (in Figs. 3B and C) to set the moment priors for Bayesian dipole fitting. This would allow us to make direct model comparison of the evidence between dipole fits based on informative priors over moment as well as location parameters. This information could also be incorporated into distributed solutions as a local current density prior. For example, at the moment it is difficult to distinguish between changes in source moment which are due to an increased local drive or an increased area of activity. Spatial models do exist (Chowdhury et al., 2013; Hillebrand and Barnes, 2011) but prior knowledge of the expected source magnitude per unit area would make these methods more robust. In this study we based our inference on data from a single time-point. We did this to keep our source model as simple as possible. Clearly we only used a very small portion of the available data and a natural extension of this approach would be to use the myelination information as additional priors on spatio-temporal dynamic causal models of the auditory system (e.g. Garrido et al., 2007; Kumar et al., 2011).

In the present study we exploited the variability of local myeloarchitecture between cortical regions, i.e., tangentially to the cortical ribbon, in order to gain increased spatial precision. Due to the excellent spatial resolution of the myelin maps (800 μm) it is in principle also possible not only to assess variability across regions, but also to resolve myelination across cortical layers (Dick et al., 2012; Sereno et al., 2013). We could thus extend the present approach by estimating myelin density at different cortical depths and assessing the predictive value of such laminar-specific myelin estimates on MEG estimates of cortical current flow. This would enable us to extend the approach presented in the current work and to identify the most likely laminar origin of the MEG signal. This is especially exciting in light of recent findings of laminar differences in gamma and alpha spike-field coherence in rhesus monkeys (Buffalo et al., 2011) and perspectives that link the laminar organisation of cortical columns with predictive coding theory (Bastos et al., 2012). In the present study we used myelin estimates to predict dipole moment strength. Extending our approach to oscillatory neuronal activity and combining those M/EEG signals with layer-resolved quantitative MPMs would provide a key link to test these laminar-specific hypotheses in humans. We could thus make non-invasive inference on brain function currently only available with invasive inter-laminar recordings.

## Figures and Tables

**Table 1:** Spearman's r correlation coefficients between dipole moments and myelin estimates. Significant correlations at an alpha level of 0.05 (uncorrected) are marked with an asterisk.

MPM\ROI	TE1.0	TE1.1	TE1.2	TE3	Patt	Post
MT	0.3818	0.3212	0.5273	0.1394	0.3091	0.4424
R1	0.5030	0.4303	0.6000*	0.4424	0.4909	0.2970
R2*	0.3576	0.5152	0.3818	0.3091	0.5879*	0.5273
